# New Prospectus of the Western Journal of the Medical and Physical Sciences

**Published:** 1837

**Authors:** Daniel Drake


### III.—ORIGINAL.

### THE WESTERN JOURNAL.

*E Sylvis Nuncius.*

CINCINNATI, OHIO, JULY, 1837.

### Art. I.—New Prospectus of the Western Journal of the Medical and Physical Sciences: owned, published and edited by the Medical Faculty of the Cincinnati College.

The undersigned begs leave to inform the profession, that he and his colleagues have become the proprietors of this, the oldest medical periodical in the Valley of the Mississippi, the eleventh volume of which is now in the press. He is happy to think, that the zeal and talents of his learned associates are likely to make the work more interesting than, in the midst of his own multiplied engagements, he has hitherto been able to render it. The editorial articles, hereafter, will be designated by the initials of their respective authors; and, as each of them is accustomed to think for himself, they will, perhaps, frequently conflict in opinion; but this circumstance will only increase the value and interest of the work.

It will comprehend—

1. Reports of cases treated in the Cincinnati Hospital, and its associate charity, the Cincinnati Eye Infirmary.

2. Original Essays, Clinical Cases, and *Post Mortem* examinations from private practice,

3. Reviews and Biographical Notices, with a quarterly catalogue of new publications.

4. Analectic articles, containing an account of new discoveries and observations, in all parts of the world where the science is cultivated.

5. The analysis of important papers in other journals.

6. Original notices and intelligence.

The matter for each volume will be arranged by an Editorial Committee: the committee for the present volume are, Drs. Harrison, Gross and Drake, to whom all communications designed for publication may be directed.

The *Publishing Committee* for this volume, are *Drs. Rives* and *Drake*, to whom all letters of business must be sent *post-paid.* The terms of publication are three dollars, if paid during the publication of the volume, or four dolllars after its completion.

In consequence of the transfer of the Journal to its present proprietors, at a late period, this number will be issued nearly a month after its proper time.

*July* 20. DANIEL DRAKE, M. D.

